# Attitudes and Perceptions of Telemedicine in Response to the COVID-19 Pandemic: A Survey of Naïve Healthcare Providers

**DOI:** 10.3389/fped.2021.647937

**Published:** 2021-04-07

**Authors:** Dana A. Schinasi, Carolyn C. Foster, M. Katie Bohling, Leonardo Barrera, Michelle L. Macy

**Affiliations:** ^1^Department of Pediatrics, Northwestern University Feinberg School of Medicine, Chicago, IL, United States; ^2^Telehealth Programs, Ann & Robert H. Lurie Children's Hospital of Chicago, Chicago, IL, United States; ^3^Mary Ann & J. Milburn Smith Child Health Outcomes, Research, and Evaluation Center, Ann & Robert H. Lurie Children's Hospital of Chicago, Chicago, IL, United States

**Keywords:** telemedicine, telehealth, COVID-19, pediatric, children's hospital, healthcare provider

## Abstract

**Introduction:** Expansion of telemedicine enabled healthcare access during the COVID-19 pandemic. In response to in-person visit restrictions, our institution trained >1,000 clinicians in telemedicine. Little is known about telemedicine-naïve pediatric healthcare provider's perceptions as they adopted telemedicine practice.

**Methods:** We conducted a cross-sectional survey of clinicians after expanding telemedicine practice at an independent children's hospital. The survey assessed experience with, concerns about, and intentions to continue telemedicine. Outpatient providers were included if they were first trained for telemedicine in response to COVID-19 and conducted at least one video visit, 3/21/2020–6/30/2020. Descriptive statistics were calculated; perceptions were compared across telemedicine activity level quartiles (based on proportions of visits delivered by video in June 2020) using Fisher's exact tests.

**Results:** Of 609 survey responses, 305 (50.1%) met inclusion criteria, representing various roles and disciplines. Over half (54.1%) conducted >20 video visits 3/21/2020–6/30/2020. More than 75% of providers found telemedicine easy to learn. Providers with greater proportions of video visits in a typical week in June reported greater ease of incorporating telemedicine into clinical practice and greater intention to continue telemedicine practice in 6 months. Nearly all providers endorsed concerns. Patient care experiences reinforced technology-related concerns and alleviated liability and privacy concerns. Payer reimbursement was the leading influencer of anticipated future use of telemedicine.

**Discussion:** Providers who conducted more telemedicine encounters reported greater ease of incorporating telemedicine into practice. Provider concerns were influenced by patient care experiences. Targeted training and quality improvement strategies are needed to sustain a robust post-pandemic telemedicine program.

## Introduction

Few pediatric providers had telemedicine experience prior to 2020 (1, 2). The COVID-19 pandemic forced clinicians to reconsider how to safely deliver care. The declaration of a national emergency in March 2020 resulted in loosening of national privacy policies and easing of state-level restrictions around provision of care and reimbursement for telemedicine (4–6). Stay-at-home orders issued to mitigate spread of COVID-19 necessitated widespread rapid deployment of telemedicine services throughout the U.S healthcare system (3). Beginning on March 21, 2020, the stay-at-home order in Illinois (7) fueled demand for telemedicine as an alternative to in-person care. Our institutional experience prior to COVID-19 aligns with previously reported barriers to telehealth adoption, including insufficient payment, inability to bill for services (2), lack of training, cost of equipment, and concerns about potential liability (8).

While Ann & Robert H. Lurie Children's Hospital of Chicago has had a dedicated telemedicine department since 2014, fewer than sixty providers had completed the required formal training to deliver care via telemedicine by the start of 2020. Telemedicine services were contained in dedicated programs that serviced unique patient populations such as neurocritical care, infectious diseases, and emergency care. The required Telemedicine Provider Training curriculum covered the foundational components of telemedicine and was tailored to provider-specific needs within each program. Before the COVID-19 pandemic, telemedicine service lines had been of limited interest to most specialties due to poor reimbursement and restrictions related to provision of telemedicine, such as the state requirement for another healthcare provider to be present at the patient site to serve as a tele-presenter. Therefore, most clinicians practicing in the outpatient settings affiliated with Lurie Children's were telemedicine-naïve, with no prior experience in this model of care delivery before the pandemic. The gubernatorial Executive Order enacted on March 19, 2020 in response to the public health emergency eased reimbursement regulations for telemedicine video and telephone visits alike.

Prior surveys of pediatric clinician attitudes on telemedicine have been conducted in similar pre-COVID settings where actual telemedicine use among providers was low. Results of a 2016 national survey on pediatricians' experiences with and attitudes toward telehealth found that 15% of pediatricians reported any telehealth use in the 12 months prior to the survey (2). With this study, we sought to examine attitudes and perceptions of those clinicians who had experience delivering care via newly adopted telemedicine practice in response to the COVID-19 pandemic. In this brief research report, we present results of a survey of telemedicine providers at our institution conducted within 3 months of their Telemedicine Provider Training and compare their attitudes relative to their self-reported telemedicine visit activity levels in June 2020. We hypothesized that attitudes and perceptions of telemedicine in the new “COVID-era” may be different as more pediatric providers have experience with telemedicine.

## Methods

### Study Design

We conducted a brief cross-sectional survey to assess perceptions of telemedicine among clinicians who completed their Telemedicine Provider Training and delivered outpatient care via video visits between March 21 and June 30, 2020. This study was deemed exempt by our Institutional Review Board.

### Setting

Ann & Robert H. Lurie Children's Hospital of Chicago is the largest independent quaternary care children's hospital in Illinois with more than 1,665 physicians and allied health professionals in 70 pediatric specialties (9). More than 220,000 children receive medical care at Lurie Children's each year, across the emergency department (ED), ambulatory, and inpatient settings. Lurie Children's Emergency Care Center is a Level 1 Pediatric Trauma Center, serving more than 56,000 ill and injured children per year. The hospital has 365 total pediatric beds, including 64 in the neonatal intensive care unit, 60 in the pediatric intensive care unit, and 12 in inpatient psychiatry.

### Telemedicine Training

Prior to being credentialed in telemedicine, all clinicians providing patient care or family support via telemedicine at our institution are required to complete formal training. Resident physicians at our institution did not participate in telemedicine during the study period. For those physicians in fellowship training, the decision to include them in their divisional telemedicine response was at the discretion of individual program leadership. Certain divisions required each of their providers to complete Telemedicine Provider Training in anticipation of use, regardless of whether explicit plans for telemedicine were yet in place. The training was delivered as a 1-h session delivered synchronously and was an institutional requirement for Telemedicine Privileges through our Medical Staff Office. Within the first 12 weeks following the declaration of national emergency, synchronous Telemedicine Provider Training was completed by 1,069 physicians, advanced practice providers, social workers, therapists, counselors, and other clinicians at Lurie Children's. The training covered foundational components of telemedicine including: local context and program goals; legal and risk considerations; high-level workflows; clinical considerations, including physical examination tips and charting requirements; virtual presence, including webside manner overview; technology overview, including hardware, software, and troubleshooting.

### Survey Development

Survey items were developed by a team experienced in telemedicine and health services research. The survey was designed to gather information about provider experiences with telemedicine, their attitudes toward telemedicine, and their intentions to continue delivering care via telemedicine. Responses from a pilot survey administered in March 2020 to previously naïve telemedicine providers at our institution were used to inform the content of this survey. The survey questions were entered into the Qualtrics XM survey platform (Qualtrics, Provo, UT). This survey was pilot tested with five individuals with expertise in evaluation and refined based on their feedback.

In the survey, “telemedicine” was defined for respondents as “real-time, face-to-face video encounter between a patient/family and a healthcare provider using a secure, HIPAA-compliant platform.” To capture outpatient clinic activity and telemedicine experience, the survey asked respondents “how many total outpatient visits did you complete in a typical week before the COVID-19 stay-at-home order,” “how many total outpatient visits did you complete in a typical week in June 2020,” and “provide your best estimate of the percentage of your outpatient visits in a typical week in June 2020 within each of the following categories”: “telephone consults,” “telemedicine (video) visits” and “in-person visits.” Respondents were asked to enter numbers totaling 100%.

To identify attitudes toward adoption of telemedicine, the survey asked “how easy or difficult it was to: (1) learn how to conduct a visit using telemedicine, and (2) incorporate telemedicine into my clinical practice;” responses were captured according to a 5-point Likert scale (“extremely easy” to “extremely difficult”). Clinicians were also queried on their intention to continue to use telemedicine in the future.

A series of questions related to concerns about telemedicine began by asking providers to select all items that cause them at least some concern from a list generated by providers at the start of the telemedicine COVID-19 response. The list of concerns included: “(1) patient privacy, (2) liability associated with telemedicine, (3) reliability of internet connections to support telemedicine, (4) families won't be able to access video services due to lack of digital devices, cellular data, or Wi-Fi, (5) limitations in the physical assessment of a patient by video, and (6) quality of audio or video will be poor.” A free-response option was also provided. For each selected item, a subsequent question asked respondents to indicate how concerned they are about each item today: “a little concerned,” “somewhat concerned,” “very concerned,” or “extremely concerned.” Respondents were also asked to indicate whether or not a patient care experience has influenced their level of concern. For each of the concerns they had selected they were provided the following response options: “I had a patient care experience that decreased this concern,” “I have not had any patient care experiences that change my concern,” and “I had a patient care experience that increased this concern.”

Providers then were asked “How did the 1-h Telemedicine Provider Training impact your overall level of concern about delivery care via telemedicine, if at all?” We also queried providers about their desire for additional training on (1) how to use the telemedicine technology, (2) webside manner or how to conduct a telemedicine visit generally, (3) how to help patient families connect through telemedicine, (4) something else with a free-text response option.

Providers were asked if they anticipated providing patient care via telemedicine in 6 months. Response options included “definitely yes,” “probably yes,” “might or might not,” “probably not,” and “definitely not;” affirmative responses (definitely or probably yes) and negative responses (definitely or probably not) were grouped for analysis. Providers were asked to rank four influencers of the continued provision of patient care via telemedicine in 6 months: division continues to offer, payers continue to reimburse, family interest, and personal preference; another response was also available. We created a categorical variable of the top influencer based on the item each provider selected as most influential. Demographic characteristics included years in clinical practice (categorical) and their area of practice/clinical background. Area of clinical practice was aggregated into the following categories: pediatric subspecialist including all medial subspecialties, general pediatrics, pediatric surgery including general surgery and surgical subspecialties, psychiatry/psychology, habilitation/rehabilitation services, clinical nutrition, genetic counseling, and other/no response.

### Survey Distribution

Anonymous survey links were distributed via email on July 8, 2020 through the hospital distribution list; reminders were sent on July 15, 2020 and targeted requests were made via email to division leadership and providers who had completed a feedback form prior to their initial training. The survey was closed to responses on August 8, 2020.

### Study Population

All 1,069 clinicians who completed formal training at our institution were considered eligible for this study. Screening questions were used to identify providers who practiced in the ambulatory setting, had not provided telemedicine care prior to March 2020 (telemedicine-naïve), and who had conducted at least one video visit in response to the COVID-19 pandemic. We excluded responses from individuals who did not provide service to patients via video visits during the period between March 21, 2020 and June 30, 2020 and those who did not progress through the entire survey.

### Analysis

Descriptive statistics were calculated including medians and interquartile ranges (IQR) for continuous data and proportions for categorical data. We characterized respondents by their telemedicine activity and categorized each provider into quartiles based on their self-reported percentage of care delivered by video visits in a typical week in June 2020. We then compared perceptions of telehealth across telemedicine activity quartiles using Fisher's exact tests. We report on perceived concerns and the change in concern based on clinical experiences, desire for additional training, intentions to continue to provide telemedicine care in 6 months, and influencers of continued provision of telemedicine. Responses were downloaded from Qualtrics and entered into Stata version 15.1 (StataCorp LLC, College Station, TX) for analysis. Free text responses to the desire for additional training were reviewed and thematically coded by one investigator (MM) and affirmed by another investigator (DS). Discrepancies were resolved through discussion.

## Results

Surveys were distributed via institution-wide and division-specific email lists. Survey links were opened by 609 staff members; 126 respondents were not eligible (93 respondents did not provide ambulatory care and 33 did not complete training). There were 483 eligible responses from 1,069 trained providers (response rate 45%). We excluded 178 surveys from our analyses (42 from providers who reported completing no video visits, 89 from providers who did not respond to the question about the number of completed telemedicine video visits, and 47 with incomplete responses to other questions relevant to our analyses). Characteristics of the 305 respondents included in the analysis and their clinical practice are presented in Table 1. The analyzed respondents represented a variety of disciplines and roles including physicians; advanced practice nurses (*n* = 43); physical therapists, occupational therapists, speech therapists (*n* = 37); social workers (*n* = 7); clinical nutritionists (*n* = 9); genetic counselors (*n* = 6); and nurses (*n* = 5). Twenty-one respondents reported that they did not complete any outpatient visits prior to the stay at home order, and 3 reported that they did not complete any visits during a typical week in June; these include academic clinicians who spend the majority of their time in research, surgeons, and other specialists who provide a mix of inpatient and outpatient care. The largest numbers of respondents reported being in practice for 15 years or more (45.3%) and conducting >20 video visits (54.1%).

**Table 1:** Respondent characteristics.

	***N =* 305**	
**Clinical role/Training**		
Pediatric subspecialist	101	33.1%
Nursing/APN	48	15.7%
PT/OT/Speech therapist	37	12.1%
Pediatric surgery	27	8.9%
Psychology	26	8.5%
General pediatrics	25	8.2%
Psychiatry	16	5.2%
Clinical nutrition	9	3.0%
Social work	7	2.3%
Other/No response	9	3.0%
**Years in practice**		
<5	61	20.0%
5–9	51	16.7%
10–14	53	17.4%
>14	138	45.3%
Missing	2	1%
**Median number of visits in a typical week prior to Stay at Home order**		
(March 21, 2020) (IQR) (*n =* 275)	15	(8, 30)
**Median number of visits in a typical week in June, 2020**		
Including in-person, telephone, and video (IQR) (*n =* 273)	15	(7, 25)
**Total number of video visits since Stay at Home order**		
*(March 21, 2020–June 30, 2020)*		
1–5	47	15.4%
6–20	93	30.5%
>20	165	54.1%
**Total number of telephone visits since Stay at Home order**		
*(March 21, 2020–June 30, 2020)*		
0	60	19.7%
1–5	89	29.2%
6–20	93	30.5%
>20	62	20.3%
Missing	1	<1%
**Proportion visits by video in a typical week in June, 2020**		
1st Quartile: <10%	75	24.6%
2nd Quartile: 10–23%	76	24.9%
3rd Quartile: 24–70%	78	25.6%
4th Quartile: >70%	76	24.9%

Respondent attitudes toward telemedicine relative to percent of visits conducted via video in a typical week in June 2020 are shown in Table 2. There were similar perceptions of ease of learning to conduct a telemedicine visit across the different quartiles of video visit activity during a typical week (*p* = 0.51). However, there was an association between the ease of incorporating telemedicine into clinical practice and the quartiles of video visit activity with 53.3% in the lowest activity quartile, 67.1 and 68.8% in the middle quartiles, and 82.9% in the highest activity quartile reporting it was very easy or easy to incorporate telemedicine into clinical practice (*p* = 0.006). Providers' intention to continue to provide care via telemedicine into the next 6 months increased incrementally by video visit activity quartile, ranging from 60.0% for the lowest activity quartile to 92.1% for the highest activity quartile (*p* < 0.0001).

**Table 2:** Attitudes toward telemedicine relative to percent of visits conducted by video in a typical week in June.

	**Quartile 1:** **<10% of visits were video**	**Quartile 2:** **10–23% of visits were video**	**Quartile 3:** **24–70% of visits were video**	**Quartile 4:** **71–100% of visits were video**	
**In your experience, how easy or difficult was it to learn how to conduct a visit using telemedicine?**					
Easy or very easy	57 (76.0%)	57 (75.0%)	62 (79.5%)	66 (86.8%)	*P =* 0.51
Neither easy nor difficult	12 (16.0%)	11 (14.5%)	12 (15.4%)	7 (9.2%)	
Difficult or very difficult	6 (8.0%)	8 (10.5%)	4 (5.1%)	3 (4.0%)	
**In your experience, how easy or difficult was it to incorporate telemedicine into your clinical practice?**					
Easy or very easy	40 (53.3%)	51 (67.1%)	53 (68.8%)	63 (82.9%)	*P =* 0.006
Neither easy nor difficult	12 (16.0%)	9 (11.8%)	10 (13.0%)	2 (2.6%)	
Difficult or very difficult	23 (30.7%)	16 (21.1%)	14 (18.2%)	11 (14.5%)	
**Thinking ahead 6 months, do you anticipate you will provide patient care via telemedicine?**					
Yes	45 (60.0%)	61 (80.3%)	71 (91.0%)	70 (92.1%)	*P <* 0.001
Unsure	18 (24.0%)	9 (11.8%)	4 (5.1%)	5 (6.6%)	
No	12 (16.0%)	6 (7.9%)	3 (3.9%)	1 (1.3%)	

The median number of concerns selected was 4 (IQR 3, 4) out of 7 possible listed concerns. Six of the 305 respondents (2.0%) selected no telemedicine concerns. Of the 299 providers indicating at least one concern, 68 (22.7%) reported that a patient care experience with telemedicine decreased their level of concern, whereas 225 (75.3%) reported that a patient care experience with telemedicine increased their level of concern. The telemedicine training curriculum decreased concerns for 101 (33.8%), increased concerns for 5 (1.7%), and had no change on concerns for 193 (64.5%).

The number of providers selecting each telemedicine-specific concern from the list of fixed-choice responses is presented in Table 3. The greatest numbers of providers selected technology-related concerns including: reliability of internet (*n* = 250, 82.0%), limitations to physical assessment by video (*n* = 225, 73.8%), family access to video services (*n* = 217, 71.1%), and poor quality of audio or video (*n* = 191, 62.6%). Less than half of responding providers reported concerns about liability (*n* = 111, 36.4%) and patient privacy (*n* = 52, 17.0%). More than half of providers who selected a technology-related concern indicated that concern had been increased by a patient care experience. Approximately 15% of providers had patient care experiences that alleviated their concerns about reliability of internet and family access to video services. Most providers had no patient care experiences that changed their liability and privacy concerns. Additional training was desired on how to help patients' families connect through telemedicine (*n* = 124), webside manner or how to conduct a telemedicine visit in generally (*n* = 48), how to use the telemedicine technology (*n* = 42), and how to document a telemedicine encounter (*n* = 41). Other training was selected by 27 and free responses included a desire for training of administrative staff to schedule telemedicine visits (*n* = 7), billing (*n* = 6), and sharing of patient education materials (*n* = 6). Free-text responses that related to technology (*n* = 10), webside manner (*n* = 5), and family support (*n* = 9) are included in the presentation of the fixed choice response results related to additional training above.

**Table 3:** Impact of patient care experience on telemedicine concerns.

**Question stem:**		**Please indicate whether or not a patient care experience has influenced your level of concern for each of the concerns you selected**.		
**Below are a list of several concerns of providers prior to our deployment of telemedicine in response to COVID-19 at Lurie Children's in mid-March, 2020. Please select the all of items that cause you at least some concern**.	N^*^ selecting concern	**Concern decreased because of patient care experience**	**No change in concern based on patient care experience**	**Concern increased because of patient care experience**
Reliability of internet connections to support telemedicine	250	36 (14.5%)	51 (20.5%)	162 (65.0%)
Limitations in the physical assessment of patient by video	225	23 (10.3%)	84 (37.5%)	117 (52.2%)
Families won't be able to access video services due to lack of digital devices, cellular data, or WiFi	217	33 (15.3%)	72 (33.3%)	111 (51.4%)
Quality of audio or video will be poor	191	9 (4.8%)	39 (20.6%)	141 (74.6%)
Liability associated with telemedicine	111	11 (9.9%)	91 (82.0%)	9 (8.1%)
Patient privacy (e.g., HIPAA and Protected Health Information)	52	10 (19.2%)	34 (65.4%)	8 (15.4%)
Other concern not listed	40	1 (2.5%)	5 (12.5%)	34 (85.0%)

* *one to two respondents did not provide responses to the change in concern based on clinical experience, resulting in a difference between the total sample size, and the sample reported in the change in concern columns*.

Most respondents (*n* = 247, 81.0%) anticipated continued practice of telemedicine 6 months after the survey, 36 respondents (11.8%) indicated they may or may not and 22 respondents (7.2%) indicated they did not anticipate continued practice of telemedicine in 6 months. Payer reimbursement was most commonly selected influencer of plans to continue the practice of telehealth (*n* = 120, 45.5%), followed by offering of telemedicine by the respondent's specialty and family preference (*n* = 62, 23.5% for each), and provider preference (*n* = 20, 7.6%). The relationship between anticipated continued telemedicine practice and selected influencers of continued practice are presented in Table 4.

**Table 4:** Top-ranked factors influencing provider intention to continue practicing telemedicine in 6 months.

			**Thinking ahead 6 months, do you anticipate you will provide care via telemedicine?**					
**Top-ranked factor influencing if you will continue to provide patient care via telemedicine 6-months from now**.	***N =* 305**	**%**	**Definitely yes or probably yes *N* = 244**	**%**	**Might or Might not *N* = 34**	**%**	**Probably not or definitely not *N* = 20**	**%**
Whether payers continue to reimburse for telemedicine visits	120	39.3%	104	46.4%	14	41.2%	2	10.0%
Whether my division continues to offer telemedicine visits	62	20.3%	56	25.0%	6	17.7%	0	0
Whether patients' families are interested in telemedicine visits	62	20.3%	46	20.5%	10	29.4%	6	30.0%
My own preference to use telemedicine in my practice	20	6.6%	10	4.5%	3	8.8%	7	35.0%
Other	14	4.6%	8	3.6%	1	2.9%	5	25.0%
No response	27	8.9%	n/a		n/a		n/a	

## Discussion

Pediatric providers previously naïve to telemedicine overwhelmingly found it easy to learn and many found telemedicine easy to incorporate into clinical practice. We found more overall positive perceptions toward telemedicine with increasing percentage of ambulatory visits conducted by video conferencing. This may signal that telemedicine becomes easier with greater use or that those who had an easier time adopting telemedicine were more likely to incorporate it into their practice. However, within this same group of providers, we identified specific ongoing concerns about telemedicine use. Specifically, providers continued to have concerns about the reliability of internet connection, the quality of video, and the limitations of physical assessment following actual patient care experiences. We also found that some providers' concerns were ameliorated through training, and that additional targeted training was desired on how to help patients' families connect through telemedicine. As a result of these findings, these areas of telemedicine delivery have become targets for education and improvement by our institution. Addressing these concerns is crucial to assuring that we provide on-going high-quality care experiences for patients and providers alike. It is important to note that the pandemic has exposed gaps in internet connectivity nationwide (10), a priority for the Federal Communications Commission to address as they strive to ensure equitable access to health care and education for all Americans.

We also found that providers who were higher utilizers of telemedicine reported greater ease of incorporating telemedicine into practice, and indicated they plan to continue its use. It is possible that some of the providers who anticipated that it would be easy to learn telemedicine were those who conducted a higher proportion of visits via telemedicine. The diffusion of innovation theory suggests that organizational structure and culture will affect health care providers' perceptions of telemedicine, thereby influencing adoption and utilization (11, 12). Similarly, the Technology Acceptance Model (TAM) connects perceived usefulness with ease of use in adoption of new technology (13). TAM is an information systems theory developed to identify how individuals begin to accept and use technological advancements, and within health care provides a better understanding of clinician technology acceptance, informing health care organizations about barriers to embracing new technologies (14). A relationship between telemedicine experience and acceptability has been previously described in providers who care for children with special health care needs (15), as well as in the tele-hospice and tele-psychiatry populations (16). Still, despite the identified relationship between technology acceptance and adoption by health care providers, there is a need to better understand the various factors contributing to this relationship (17). As such, our survey results support the need for a more targeted framework to better define this relationship. Numerous medical education frameworks already exist for achieving mastery of essential clinical skills (18, 19), and the field of telemedicine is poised for the merging of technology and education frameworks to achieve this.

Our study has several limitations. Our survey was conducted at a single center; therefore, findings may not be generalizable to settings with different telemedicine training or platforms. Surveys were distributed via mass communication channels to all staff members, including those who do not provide clinical care and those who were not trained in the telehealth pandemic response. We also distributed surveys with anonymous links. Responses represent a subset of individuals who completed telemedicine training and there is potential for response bias. We did not have a mechanism to obtain information from non-respondents and therefore cannot determine if respondents differ from non-respondents. It is possible that providers who completed the survey were representative of the full population. It is also possible that respondents had stronger opinions, either positive or negative, toward telemedicine than non-respondents, but we do not have a way to quantify the impact of response bias on our results. Because of skip and display logic, we do not have a way to compare providers who completed video visits to those who did not. Surveys are also subject to social desirability bias. This bias is minimized by allowing providers to provide anonymous survey responses. Additionally, while all providers were uniformly trained in telemedicine with consistency in standards and technology platforms, integration of telemedicine into ambulatory workflows was at the discretion of individual divisions, some of whom have a medical assistant or nurse to help the provider and others who do not. This data was not collected in our study and is thus a limitation. There are myriad unmeasured factors that could have influenced the ease with which a provider was able to incorporate telemedicine into their practice. This is an area for future research.

The global pandemic exposed providers and patients to telemedicine, many of whom were previously naïve to this modality of care delivery. Our findings support the acceptability of telemedicine in outpatient pediatric care and suggest that with increased experience telemedicine becomes easier to incorporate into practice. Targeted training and quality improvement strategies are needed to sustain a robust post-pandemic telemedicine program. Provider concerns about telemedicine were both reinforced and alleviated by patient care experiences; this lack of distinct directionality is a precursor for future qualitative work, to better describe how provider concerns with telemedicine are either reinforced or alleviated in relation to the patient experience in order to identify areas for additional support. Telehealth programs further can address provider concerns through advocacy for policy change and investment in resources to ensure patients have access to technology needed to utilize telemedicine services.

## Data Availability Statement

The raw data supporting the conclusions of this article will be made available by the authors, without undue reservation.

## Ethics Statement

Ethical review and approval was not required for the study on human participants in accordance with the local legislation and institutional requirements. The patients/participants provided their written informed consent to participate in this study.

## Author Contributions

DS conceptualized the study, designed the data collection instrument, drafted the initial manuscript, and reviewed and revised the manuscript. CF conceptualized the study, designed the data collection instrument, and reviewed and revised the manuscript. MB conceptualized the study, designed the data collection instrument, and critically reviewed the manuscript for important intellectual content. LB carried out the data analysis and reviewed and revised the manuscript. MM conceptualized the study, designed the data collection instrument, supervised data collection, carried out the data analysis, and reviewed and revised the manuscript. All authors approved the final manuscript as submitted and agree to be accountable for all aspects of the work.

## Conflict of Interest

The authors declare that the research was conducted in the absence of any commercial or financial relationships that could be construed as a potential conflict of interest.
